# Coinfections and antimicrobial treatment in a cohort of falciparum malaria in a non-endemic country: a 10-year experience

**DOI:** 10.1007/s15010-023-02103-x

**Published:** 2023-10-27

**Authors:** Claus P. Küpper-Tetzel, Raja Idris, Johanna Kessel, Gundolf Schüttfort, Sebastian Hoehl, Niko Kohmer, Christiana Graf, Michael Hogardt, Silke Besier, Thomas A. Wichelhaus, Maria J. G. T. Vehreschild, Christoph Stephan, Nils Wetzstein

**Affiliations:** 1 Department of Internal Medicine, Infectious Diseases, University Hospital Frankfurt, Goethe University, Theodor-Stern-Kai 7, 60590 Frankfurt, Germany; 2 Institute of Medical Virology, University Hospital Frankfurt, Goethe University, Frankfurt am Main, Germany; 3 Department of Internal Medicine, Gastroenterology and Hepatology, University Hospital Frankfurt, Goethe University, Frankfurt am Main, Germany; 4 Institute of Medical Microbiology and Infection Control, University Hospital Frankfurt, Goethe University, Frankfurt am Main, Germany

**Keywords:** Falciparum malaria, Malaria, *Plasmodium falciparum*, Antibiotic stewardship, Returning travellers, Visiting friends and relatives

## Abstract

**Introduction:**

Falciparum malaria remains one of the deadliest infectious diseases worldwide. In Germany, it is mainly an imported infection among travellers. Rates of coinfection are often unknown, and a clinical rationale for the beneficial use of calculated antibiotic therapy in patients with malaria and suspected coinfection is lacking.

**Methods:**

We conducted an analysis of all in-patients treated with falciparum malaria at a German infectious diseases centre in vicinity to one of Europe’s major airports for 2010–2019. Logistic regression and time-to-event analysis were used to evaluate predictors for bacterial coinfection, the use of antibacterial substances, as well as their influence on clinical course.

**Results:**

In total, 264 patients were included. Of those, 64% received an additional antibacterial therapy (*n* = 169). Twenty-nine patients (11.0%) were found to have suffered from a relevant bacterial coinfection, while only a small fraction had relevant bacteremia (*n* = 3, 1.4%). However, patients with severe malaria did not suffer from coinfections more frequently (*p* = 0.283). CRP levels were not a reliable predictor for a bacterial coinfection (OR 0.99, 95% CI 0.94–1.06, *p* = 0.850), while another clinical focus of infection was positively associated (OR 3.86, 95% CI 1.45–11.55, *p*  = 0.010).

**Conclusion:**

Although bacterial coinfections were rare in patients with malaria at our centre, the risk does not seem negligible. These data point rather towards individual risk assessment in respective patients than to general empiric antibiotic use.

## Introduction

Falciparum malaria (malaria) remains one of the deadliest infectious diseases worldwide and is caused by *Plasmodium falciparum* [1]. The disease was eradicated from Europe by the mid-twentieth century and at present occurs nearly exclusively as imported cases or imported infected mosquitos in non-endemic countries [2, 3]. In Germany, the number of malaria cases reported to the Robert Koch Institute ranged from 617 in 2010 to 1068 in 2015 to 993 in 2019. The rise in cases started in 2014 and is largely attributed to increased migration from Africa [4–6]. Returning travellers, immigrants, as well as those visiting friends and relatives (VFR) account for the majority of cases in non-endemic countries [7]. The latter are especially at risk, because there are misconceptions about a lifelong immunity against malaria and antimalarial prophylaxis might not be considered necessary [8]. In addition to these routes of imported infections, local transmission in the vicinity of airports (so called airport malaria) is possible and has been described for the Frankfurt area [9], and among other places [10].

In Europe, the most frequent causes of fever in returning travellers are malaria and dengue virus infection, followed by bacterial infections [11]. Conventional inflammation markers such as C-reactive protein (CRP) and procalcitonin (PCT) can be elevated in patients with bacterial infections, but also in falciparum malaria, rendering their discrimination a clinical challenge [12]*.* Besides fever, patients with malaria often present with a variety of unspecific clinical signs that might be attributed to other imported infections, such as pneumonia or bacterial diarrhea [13].

In addition to antimalarial treatment, WHO guidelines advise for empirical antibiotic treatment in severely ill children in malaria-endemic areas as bacterial meningitis and severe falciparum malaria are both common in endemic areas [1]. However, it is generally not recommended to adapt the same strategy in adults, the WHO guidelines recommend the use of empirical broad-spectrum antibiotics in cases where bacterial meningitis cannot be ruled out or when there is clear evidence of aspiration [1]. German guidelines for the treatment of malaria advocate the consideration of empiric antibiotic therapy in those with symptoms sepsis or shock [14]. Prior studies have shown that there seems to be a low incidence of bacterial coinfections in returning travellers with falciparum malaria [15]*.* Nevertheless, the clinician might tend to use an antibacterial agent in those patients, guided by laboratory parameters and clinical picture. However, unnecessary use of antibiotics can have unwanted effects, such as the development of multidrug-resistant organisms (MDRO) [16], or an increase in length of hospital stays [17], and should be avoided.

Therefore, the aims of this study were to (i) assess the prevalence of bacterial, viral and parasitic coinfections in patients with malaria treated in a German infectious disease centre in vicinity to one of the main European airports, and (ii) to explore the influence of calculated antibiotic therapies on the clinical course in those patients.

## Methods

### Inclusion criteria and clinical data

This retrospective cohort study was approved by our local ethics committee under file number 20-1009. A data base query with malaria-related ICD codes B50-B54 was conducted in our local patients’ database for the time period 2010–2019.

Frankfurt University Hospital is a regional centre for the treatment of imported malaria infections, especially due to its vicinity to Frankfurt International Airport. All in-patients over 18 years of age suffering from infection due to *Plasmodium falciparum,* i.e. falciparum malaria, were included. Individuals with infections due to *Plasmodium vivax*, *Plasmodium ovale* and *Plasmodium malariae* were excluded from the analysis, as well as patients that were only treated as outpatients.

Three infectious disease physicians (CPKT, RI and NW) retrieved patient data by chart review in our local patient data base system (ORBIS, Dedalus Healthcare Systems Group, Bonn, Germany). The following baseline parameters were recorded: patients’ ages and sex; country of origin and travel country; antimalarial and antibacterial treatment; duration of fever and duration of hospitalisation; admission to the intensive care unit and death; and possible other clinical focus of infection.

During the first 5 days of treatment (if applicable) we observed the following laboratory and clinical parameters: C-reactive protein (CRP, [mg/dl]), hemoglobin (Hb, [g/dl]), platelet count (plt, [/nl]), white blood cell count (WBC, [/nl]), as well as maximum corporeal temperatures for each day and time to defervescence. Severe malaria was defined according to WHO definitions from 2021 [1].

### Microbiological assessment

Malaria was diagnosed by rapid antigen tests (BinaxNOW Malaria Test, Abbott, Chicago, USA), fluorescent microscopy (quantitative buffy coat – QBC Malaria Tubes, QBC Diagnostics, Philipsburg, USA), as well as blood smear colored by Giemsa stain (Merck, Darmstadt, Germany) by infectious diseases physicians at University Hospital Frankfurt (CPKT, JK, GS, TW, CS, NW). Initial parasite density was measured directly at admission in the blood smear.

To assess the occurrence of coinfections, we recorded the following microbiological and virological examinations and their results: blood cultures, urine cultures, dengue virus serology, chikungunya virus serology, Zika virus serology, serological tests for *Legionella* spp., *Mycoplasma* spp. and *Chlamydia* spp. If other microbiological examinations turned out positive, they were recorded as well. If microbial pathogens other than *Plasmodium falciparum* were detected in those examinations, their clinical relevance was rated independently according to plausibility, the probability of contamination or cross-reactions by two physicians (CPKT and NW). If incongruent ratings occurred, discussion led to a consensus decision in individual cases. If there was suspicion of pneumonia, X-ray of the chest was performed. Radiological findings were included in the analysis.

### Statistical analysis

Data were recorded in Microsoft Excel and finally analysed in R Version 4.2.1. “*Vigorous calisthenics*” [18]. Categorical data are depicted in numbers and percentages, continuous data as median with interquartile range (IQR) for non-normally distributed data and mean with range for normally distributed data. We used the Shapiro–Wilk test to test for normality. All clinical data and microbiological results were stratified by severe and non-severe malaria. Geographical maps were drawn using the *ggmap* and *ggplot2* packages [19, 20]. Coordinates of the country’s capital in which the infection was acquired or the country’s centroid were used as a reference. Time-to-event analysis was conducted using *survival* and *survminer* packages within R [21, 22]. Uni- and multivariate logistic regression were performed in R using the *finalfit* package [23] with the dependent variable being bacterial coinfection/the use of antibiotic substances and following parameters: parasite density, Hb, thrombocytes and leucocytes, age, gender, clinical focus, X-ray results, urine culture results, severe malaria, ICU stay and defervescence.

## Results

### Included patients and general characteristics

In total, 264 patients with confirmed falciparum malaria were included. Of those, 65.9% (*n* = 174) were male and 34.1% (*n* = 90) female (Table 1). Median age was 40 years at the time of infection (IQR 30–50 years). 18.6% of patients fulfilled the WHO criteria for severe malaria (n = 49), with a bilirubin level above 3 mg/dl being the criterion most frequently met (*n* = 29, 59.2%). 10.6% of patients were admitted to the IMC/ICU (*n* = 28) and 1.1% of patients deceased (*n* = 3). Patients suffered from a median temperature of 38.9 °C at day one of admission (IQR 37.6–39.5 °C). On the same day the median CRP count was 8.3 mg/dl (IQR 5.0–12.3 mg/dl), hemoglobin 13.2 g/dl (IQR 11.7–14.7 g/dl), platelets at 86/nl (IQR 48.8–129.3/nl) and white blood cell count (WBC) at 4.95/nl (IQR 4.1–6.2/nl).

**Table 1:** Baseline characteristics of included patients

Categorical variables	All patients (*n* = 267)	Severe malaria (*n* = 49)	Non-severe malaria (*n* = 215)	*p* value
	*n*/*N* (%)	*n*/*N* (%)	*n*/*N* (%)	
Gender				
Male	174/264 (65.9)	30/49 (61.2)	144/215 (67.0)	0.5047
Female	90/264 (34.1)	19/49 (38.8)	71/215 (33.0)	0.5047
Complicated malaria	49/264 (18.6)	49/49 (100)	0/215 (0)	NA
Cerebral	16/49 (32.7)	16/49 (32.7)	NA	NA
Prostration	25/49 (51)	25/49 (51)	NA	NA
Convulsions	1/49 (2)	1/49 (2)	NA	NA
Acidosis	8/49 (16.3)	8/49 (16.3)	NA	NA
Hypoglycaemia	1/49 (2)	1/49 (2)	NA	NA
Hb < 5 mg/dl	1/49 (2)	1/49 (2)	NA	NA
Renal impairment	20/49 (40.8)	20/49 (40.8)	NA	NA
Bilirubin > 3 mg/dl	29/49 (59.2)	29/49 (59.2)	NA	NA
Pulmonary edema	11/49 (22.4)	11/49 (22.4)	NA	NA
Significant bleeding	3/49 (6.1)	3/49 (6.1)	NA	NA
Shock	12/49 (24.5)	12/49 (24.5)	NA	NA
Parasitic density > 10%	12/49 (24.5)	12/49 (24.5)	NA	NA
Relevant coinfection	41/264 (15.5)	10/49 (20.4)	31/215 (14.4)	0.283
Bacterial	29/264 (11.0)	8/49 (16.3)	21/215 (9.8)	0.2058
Viral	14/264 (5.3)	3/49 (6.1)	11/215 (5.1)	0.7285
Parasitic	1/264 (0.4)	0/49 (0)	1/215 (0.5)	1
Admitted to IMC/ICU	28/264 (10.6)	23/49 (46.9)	5/215 (2.3)	** < 0.0001**
Deceased	3/264 (1.1)	3/49 (6.1)	0/215 (0)	** < 0.01**

Continuous variables	Median (IQR)	Median (IQR)	Median (IQR)	
Age [years]	40 (30–50)	41 (31–55)	40 (29–50)	0.108
Hospitalisation [d]	5 (4–6)	7 (5–11)	4 (4–5)	** < 0.0001**
Time to defervescence [d]	2 (1–2)	2 (1–3)	2 (1–2)	< 0.05
Parasital density at d1 [%]	1 (0.2–3.05)	5 (3–10)	0.8 (0.2–1.75)	** < 0.0001**
Fever at d1 [°C]	38.9 (37.6–39.5)	38.5 (37.6–39.6)	38.9 (37.6–39.4)	0.6827
CRP at d1 [mg/dl]	8.345 (4.99–12.275)	13.43 (7.66–18.06)	7.68 (4.5–11.4)	** < 0.0001**
Hb at d1 [g/dl]	13.2 (11.7–14.7)	12.3 (10.6–13.6)	13.3 (11.9–13.2)	** < 0.01**
Platelets at d1 [/nl]	86 (48.75–129.25)	45 (29–79)	102 (60.5–137.5)	** < 0.001**
WBC at d1 [/nl]	4.945 (4.065–6.232)	5.78 (4.41–8.54)	4.84 (4.0–6.0)	** < 0.01**

Significant *p* values are marked in bold

*Hb* hemoglobin, *IMC* intermediate care, *ICU* intensive care unit, *CRP* C-reactive protein, *WBC* white blood cell count, *NA* not applicable

The majority of patients acquired their infection on the African continent (*n* = 259, 98.1%, Fig. S1) with Ghana (*n* = 43), Nigeria (*n* = 43), Cameroon (*n* = 36) and Togo (*n* = 24) being the countries from which travellers returned most frequently. Only five patients were infected in other parts of the world: two patients in the Dominican Republic, one in Papua New Guinea and finally two cases of airport malaria in Frankfurt, Germany that have been described elsewhere [9].

### Antimicrobial therapy and coinfections

All patients received antimalarial treatment with either artemether/lumefantrine (*n* = 234, 88.6%), atovaquone/proguanil (*n* = 41, 15.5 percent), artesunate (*n* = 26, 9.8%), quinine (*n* = 4, 1.5%) or chloroquine (*n* = 2, 0.8%) (Table 2). Patients initially treated with intravenous artesunate received oral follow-up treatment with one of the above-mentioned agents. Overall, 64% of the patients received an additional antibacterial therapy (*n* = 169). A vast proportion of patients with severe malaria received antibiotic therapy (89.8%, *n* = 44). In contrast, only 58.1% (*n* = 125) of patients with non-severe malaria received antibacterial therapy. Cephalosporins were most frequently administered (*n* = 120, 71% of patients receiving antibacterial therapy), followed by carbapenems (*n* = 31, 18.3%), fluoroquinolones (*n* = 28, 16.6%) and metronidazole (*n* = 23, 13.6%).

**Table 2:** Administered antimicrobial therapy, alternative clinical focus and apparative diagnostics

	All patients (*n* = 264)	Severe malaria (*n* = 49)	Non-severe malaria (*n* = 215)	*p* value
	*n*/*N* (%)	*n*/*N* (%)	*n*/*N* (%)	
Antimalarial treatment	264/264 (100)	49/49 (100)	215/215 (100)	1
Artemether/lumefantrine	234/264 (88.6)	35/49 (71.4)	199/215 (92.6)	** < 0.001**
Atovaquone/proguanil	41/264 (15.5)	18/49 (36.7)	23/215 (10.7)	** < 0.0001**
Artesunate (i.v.)	26/264 (9.8)	23/49 (46.9)	3/215 (1.4)	** < 0.0001**
Chloroquine	2/264 (0.8)	1/49 (2.0)	1/215 (0.5)	0.3373
Quinine	4/264 (1.5)	4/49 (8.2)	0/215 (0)	** < 0.01**
Antibiotic treatment	169/264 (64)	44/49 (89.8)	125/215 (58.1)	** < 0.0001**
Cephalosporin	120/169 (71)	25/44 (56.8)	95/125 (76)	** < 0.05**
Acylaminopenicilline	15/169 (8.9)	8/44 (18.2)	7/125 (5.6)	** < 0.05**
Carbapenem	31/169 (18.3)	15/44 (34.1)	16/125 (12.8)	** < 0.05**
Fluoroquinolone	28/169 (16.6)	9/44 (20.5)	19/125 (15.2)	0.4803
Doxycycline	22/169 (13)	9/44 (20.5)	13/125 (10.4)	0.1166
Metronidazole	23/169 (13.6)	8/44 (18.2)	15/125 (12)	0.3137
Others	23/169 (13.6)	11/44 (25)	12/125 (9.6)	** < 0.05**
Other clinical focus	107/264 (40.5)	31/49 (63.3)	76/215 (35.3)	** < 0.001**
Pulmonary	29/107 (27.1)	6/31 (19.4)	23/76 (30.3)	0.339
Diarrhea	61/107 (57)	19/31 (61.3)	42/76 (55.3)	0.6684
Cerebral	9/107 (8.4)	8/31 (25.8)	1/76 (1.3)	** < 0.001**
Urinary	10/107 (9.3)	2/31 (6.5)	8/76 (10.5)	0.7203
Others	16/107 (15)	4/31 (12.9)	12/76 (15.8)	1
Apparative diagnostics				
Radiograph of thorax	47/208 (22.6)	14/41 (34.1)	33/167 (19.8)	0.06066
Urine dipstick	11/187 (5.9)	2/40 (5)	9/147 (6.1)	1
MDRO screening	218/264 (82.6)	42/49 (85.7)	176/215 (81.9)	0.6769
Multi-drug resistant gram negatives	38/219 (17.4)	12/42 (28.6)	16/176 (9.1)	** < 0.05**
VRE	3/219 (1.4)	2/42 (4.8)	1/176 (0.6)	0.09572
MRSA	0/219 (0)	0/42 (0)	0/176 (0)	1

Significant *p* values are marked in bold

*MDRO* multi-drug resistant gram organisms, *VRE* vancomycin-resistant enterococci, *MRSA* methicillin-resistant *Staphylococcus aureus*

^a^These organisms have been classified according to the KRINKO definition (doi: 10.25646/5916)

Clinical symptoms concordant with a possible alternative focus were found in 40.5% of patients (*n* = 107), with diarrhea (*n* = 61, 57%) and pulmonary symptoms (e.g. cough, *n* = 29, 27.1%) being the most frequent clinical signs. Among patients that underwent screening for multi-drug resistant gram-negative bacteria, 17.4% had positive swabs (*n* = 38).

Interestingly, serological tests for Zika virus were positive in 44.2% of patients in whom they were performed (19 out of 43, Table S1). However, PCR was performed in six patients of which none turned out positive and no seroconversion could be observed (Table S5). 1.5% of patients (and 2.8% of performed tests) were shown to suffer from concomitant dengue virus infection. Of those, one patient died directly after admission (most likely due to a pronounced parasite density). Furthermore, no positive serologies for *Legionella pneumophila* (including urine antigen tests) or *Chlamydia pneumoniae* were detected, whereas *Mycoplasma pneumoniae* serologies turned out positive (IgM) in 20.8% of cases (5 out of 24).

In only 3 out of 219 patients with blood cultures clinically relevant pathogens were cultivated (1.4% of patients with blood cultures, Tables S1 and S2): *Escherichia coli, Salmonella chester*, *Streptococcus oralis/mitis* and *salivarius.* The last two pathogens were found in blood cultures from the same patient. Ten out of 161 urine cultures (6.2%) were rated to have contained pathogens responsible for urinary tract infection:* Escherichia coli* (8 cases), *Enterococcus faecalis* (3 cases), as well as *Citrobacter* spp., *Pseudomonas aeruginosa* and *Klebsiella pneumoniae* (one case each).

41 patients were rated to suffer from a relevant coinfection (15.5% of all patients). Fourteen patients (5.3%) were classified with a viral, 29 (11.0%) with a bacterial and one (0.4%) patient with a parasitic coinfection. Of those, three patients were suffering from concomitant viral and bacterial coinfection. There were no significant differences between patients with severe or non-severe malaria within all three categories (Table 1). Ten out of 49 patients with severe malaria presented with a coinfection. Of those, eight were bacterial (Table 1). Of all patients admitted to the ICU (*n* = 28), seven had a coinfection, of which four were bacterial.

### Clinical course

**Fig. 1:**
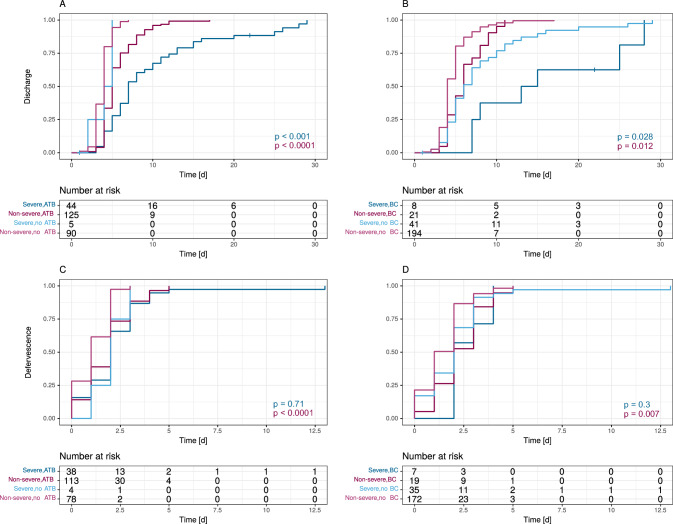
Time to event data for discharge (**A**, **B**) and time to defervescence (**C**, **D**) stratified by the reception of antibiotic treatment and a confirmed bacterial coinfection, as well as the occurrence of severe and non-severe malaria. Number at risk corresponds to the number of patients available for analysis within the respective observation period. *ATB* received antibiotic treatment, *BC* bacterial coinfection

**Fig. 2:**
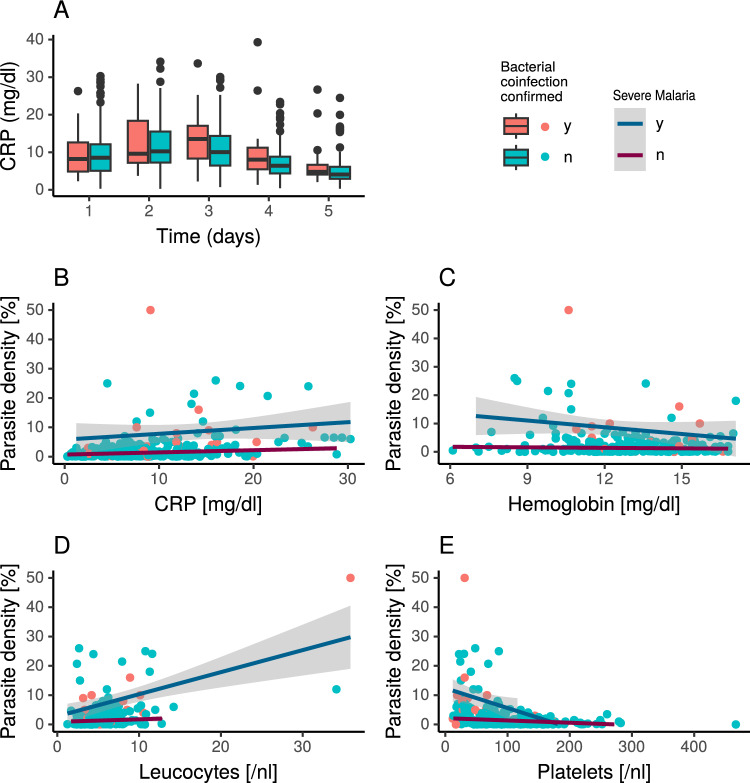
CRP values for days 1–5 stratified by the confirmation of a bacterial coinfection (**A**), as well as correlation between parasite density and CRP, hemoglobin, leucocytes and platelets (**B**–**E**)

The median hospitalisation period was 5 days (IQR 4–6 days) and the median time to defervescence 2 days (IQR 1–2 days). Unsurprisingly, 46.9% of patients with severe malaria were treated in the IMC/ICU, but only 2.3% of patients with non-severe malaria (Table 1). An antibiotic treatment was associated with a later discharge in the time-to-event analysis both for patients with severe and non-severe malaria (Fig. 1A, *p* < 0.001 and *p* < 0.0001, respectively), while it was significantly associated with longer periods to defervescence in patients with non-severe malaria (Fig. 1C, *p* < 0.0001). The same was the case for a confirmed bacterial coinfection (Fig. 1B, D). C-reactive protein levels were significantly higher on all days of treatment in patients who received antibacterial therapy than in those that did not (*p* < 0.001 for days 1–5) suggesting an elevated CRP level being a trigger for antibiotic therapy. However, patients with a confirmed bacterial coinfection did not show elevated CRP levels in comparison to those without (Fig. 2A, *p* = 0.85 on day 1, *p* = 0.35 on day 2, *p* = 0.08 on day 3, *p* = 0.10 on day 4, and *p* = 0.24 on day 5).

On the other hand, CRP levels on day 1 were positively associated with parasite density (Fig. 2B, *p* < 0.001 in univariate analysis and *p* = 0.11 in multivariate analysis), as well as leucocyte count (Fig. 2D, p < 0.001 in univariate analysis and *p* < 0.001 in multivariate analysis). Hemoglobin levels and platelet counts on day 1 of admission were negatively associated with parasite density (Fig. 2C, E,  *p* = 0.003/*p* = 0.012 and *p* < 0.001/*p* < 0.001, respectively). Of only 12 patients with distinct leukocytosis (> 10.41/nl), two had a bacterial coinfection with 10.52/nl and 35.84/nl, respectively.

Overall, a confirmed coinfection (either viral, bacterial or parasitic) was positively associated with the administration of an antibacterial treatment (empiric or targeted) in the multivariate analysis (OR 4.37 95% CI 1.26–20.73, *p* = 0.033), followed by a positive radiograph of the thorax (performed in 208 cases and yielding positive results in 47 patients, 22.6%) (OR 3.41 CI 1.17–11.60, *p* = 0.028) (Table S3). On the other hand, the latter was not a significant positive predictor for a bacterial coinfection (OR 2.32, 95% CI 0.91–5.64, *p* = 0.067), while another clinical focus of infection was (OR 3.86, 95% CI 1.45–11.55, *p* = 0.01) (Table 3).

**Table 3:** Univariate and multivariate logistic regression for the occurrence of bacterial coinfection

Variable	Factor	Bacterial coinfection			
		y	*n*	Univariate analysis	Multivariate analysis
				OR (95% CI, *p*value)	OR (95% CI, *p*value)
Gender	f	17 (18.9)	73 (81.1)	3.14 (1.44–7.07, *p* = 0.004)	2.71 (1.03–7.38, *p* = 0.045)
	m	12 (6.9)	162 (93.1)	–	–
Other clinical focus	y	21 (19.6)	86 (80.4)	4.55 (2.00–11.34, *p* = 0.001)	3.86 (1.45–11.55, *p* = 0.010)
	n	8 (5.1)	149 (94.9)	–	–
Result of chest radiograph	pos	9 (19.1)	38 (80.9)	2.32 (0.91–5.64, *p* = 0.067)	–
	neg	15 (9.3)	147 (90.7)	–	–
Result of urine dipstick	pos	4 (36.4)	7 (63.6)	4.00 (0.98–14.40, *p* = 0.038)	2.49 (0.42–12.98, *p* = 0.282)
	neg	22 (12.5)	154 (87.5)	–	–
Severe malaria	y	8 (16.3)	41 (83.7)	1.80 (0.71–4.22, *p* = 0.190)	–
	n	21 (9.8)	194 (90.2)	–	–
Admission to ICU	y	4 (14.3)	24 (85.7)	1.41 (0.39–4.01, *p* = 0.556)	–
	n	25 (10.6)	211 (89.4)	–	–
Age	Mean (SD)	41.3 (14.1)	40.6 (12.8)	1.00 (0.97–1.03, *p* = 0.797)	–
Parasital density at d1 [%]	Mean (SD)	5.1 (9.6)	2.4 (4.4)	0.94 (0.89–0.99, *p* = 0.029)	1.01 (0.93–1.12, *p* = 0.837)
Time to defervescence [d]	Mean (SD)	2.5 (1.2)	1.6 (1.4)	0.71 (0.53–0.92, *p* = 0.016)	0.84 (0.63–1.12, *p* = 0.205)
Fever at d1 [°C]	Mean (SD)	38.9 (1.1)	38.4 (2.9)	0.79 (0.53–1.06, *p* = 0.210)	–
CRP at d1 [mg/dl]	Mean (SD)	9.5 (6.0)	9.3 (6.0)	0.99 (0.94–1.06, *p* = 0.850)	–
WBC at d1 [/nl]	Mean (SD)	6.5 (6.0)	5.4 (2.8)	0.94 (0.86–1.03, *p* = 0.136)	–

## Discussion

This study investigates a cohort of patients with falciparum malaria treated at a central German infectious disease centre in the vicinity of one of Europe’s major airports. The cohort was mainly composed of returning travellers that acquired their infection in Africa.

Only 18.6% (*n* = 49) of patients fulfilled WHO criteria for complicated malaria and 10.6% (*n* = 28) were admitted to the IMC/ICU. Twenty-nine patients suffered from a relevant bacterial coinfection, whereas clinically relevant bacteremia occurred only in a small fraction of patients (1.4% of patients with blood cultures). The results are in line with those from other European countries: relevant bacteremia in returning travellers with diagnosed malaria seems to be a rare event [15]. However, the high rate of antibacterial therapy in our cohort might bias these results because we could not retrace whether blood cultures and other bacterial cultures were taken before or after the first administration of an antibiotic agent.

Sixty-four percent of patients received an antibacterial therapy that was mainly guided by positive radiographs of the thorax, as well as high CRP levels. However, CRP counts were not significantly higher in patients with confirmed bacterial coinfection, than in those without. Moreover, CRP levels correlated with parasite density at day one and they were significantly higher in the severe malaria group (p < 0.0001). These results confirm prior studies, in which the measurement of CRP alone was not sufficient to discriminate between a bacterial infection and malaria [12]***.*** We identified a comparable European cohort of 765 febrile international travellers: here, elevated CRP levels were independently associated with malaria. Patients with viral cause of fever had a CRP level of 1 mg/dl, those with bacterial acute undifferentiated febrile illness 3.5 mg/dl and undiagnosed acute undifferentiated febrile illness 2.5 mg/dl compared with a CRP of 9.1 mg/dl in malaria patients [11].

Unfortunately, PCT was not routinely measured in our cohort, therefore, we could not systematically compare this value between both groups. Interestingly, of patients with a bacterial coinfection only two exhibited a significant leukocytosis. In regards to the radiological results, microsequestration of *Plasmodium falciparum* provokes respiratory distress in 25% of adult patients and 40% of children and can even lead to acute respiratory distress syndrome (ARDS) as a severe complication [24, 25]. Therefore, a majority of infiltrates observed in radiographs of our patients might have been attributed to malaria alone and did not justify antibacterial therapy. The fact, that serologies for bacteria causing atypical pneumonia (such as chlamydia, mycoplasma, and legionella) were frequently performed, but rarely delivered significant results, emphasizes this.

Interestingly, 1.5% of patients (and 2.8% of performed tests) were shown to suffer from concomitant dengue virus infection, warranting the practice of its detection in the presence of a corresponding clinical picture. Serological tests for Zika virus were performed in view of the Zika epidemic 2007–2019 [26], 16.3% of patients (and 32.6% of performed tests) were serologically reactive for Zika virus. However, PCR was performed in six patients of which none turned out positive and confirmatory tests were negative in all cases. Our results confirm other studies demonstrating false-positive Zika virus serologies especially in patients with high malaria parasite loads [27–29]**.** Polyclonal B-lymphocyte stimulation might be a reason for positive serological tests in patients with Malaria [30]. If Zika virus coinfection is suspected in malaria patients, PCR tests instead of serological tests are advisable.

One of the main limitations of this study is its monocentric and retrospective nature. Therefore, some vital information such as the exact time of acquiring samples for bacterial cultures could not be assessed. This might have led to a reduced detection of bacterial pathogens in patients receiving an antibacterial therapy prior to sample collection. This might be especially the case in patients with severe malaria, as those received an additional antibiotic therapy more frequently. Furthermore, additional parameters such as PCT or other virological and microbiological examinations than those assessed could not be analysed. Still, our 264 travellers returning with malaria remain a cohort of considerable size for Germany and comprises a substantial share of malaria patients in Germany (*Table S4*).

## Conclusion

Empiric antibiotic therapy in returning travellers with falciparum malaria should not be guided by CRP levels or radiographs of the thorax alone. Although bacterial coinfections were rarely detected in patients with malaria at our centre, the risk does not seem negligible. These data point rather towards individual risk assessment in respective patients than to the general use of empirical antibiotic therapy.

## Data Availability

All relevant data are presented in the manuscript. More detailed data can be provided by the authors upon reasonable request.
